# Different meaning of the mean heart dose between 3D-CRT and IMRT for breast cancer radiotherapy

**DOI:** 10.3389/fonc.2022.1066915

**Published:** 2023-01-16

**Authors:** Jessica Prunaretty, Celine Bourgier, Sophie Gourgou, Claire Lemanski, David Azria, Pascal Fenoglietto

**Affiliations:** ^1^ Institut de Recherche en Cancérologie de Montpellier (IRCM), INSERM U1194, Montpellier, France; ^2^ Fédération Universitaire d’Oncologie Radiothérapie d’Occitanie Méditerranée, Institut régional du Cancer Montpellier (ICM), Montpellier, France; ^3^ Université Montpellier, Montpellier, France; ^4^ Biostatistics Department, Institut du Cancer de Montpellier, Montpellier, France

**Keywords:** breast cancer radiotherapy, heart exposure, IMRT, 3D-CRT, mean heart dose

## Abstract

**Background:**

Previous studies in 2D and in 3D conformal radiotherapy concludes that the maximal heart distance and the mean heart dose (MHD) are considered predictive of late cardiac toxicities. As the use of inverse-planned intensity modulated radiation therapy (IMRT) is increasing worldwide, we hypothesized that this 3D MHD might not be representative of heart exposure after IMRT for breast cancer (BC).

**Methods:**

Patients with left-sided BC and unfavorable cardiac anatomy received IMRT. Their treatment plan was compared to a virtual treatment plan for 3D conformal radiotherapy with similar target volume coverage (study A). Then, a second 3D conformal treatment plan was generated to achieve equivalent individual MHD obtained by IMRT. Then the heart and left anterior descending (LAD) coronary artery exposures were analyzed (study B). Last, the relationship between MHD and the heart volume or LAD coronary artery volume receiving at least 30Gy, 40Gy and 45Gy in function of each additional 1Gy to the MHD was assessed (study C).

**Results:**

A significant decrease of heart and LAD coronary artery exposure to high dose was observed with the IMRT compared with the 3D conformal radiotherapy plans that both ensured adequate target coverage (study A). The results of study B and C showed that 3D MHD was not representative of similar heart substructure exposure with IMRT, especially in the case of high dose exposure.

**Conclusions:**

The mean heart dose is not a representative dosimetric parameter to assess heart exposure following IMRT. Equivalent MHD values following IMRT and 3DRT BC treatment do not represent the same dose distribution leading to extreme caution when using this parameter for IMRT plan validation.

## Background

Breast-conserving surgery followed by whole breast irradiation (WBI) is the current standard of care for patients with early stage breast cancer (BC). Although WBI significantly decreases the risk of locoregional recurrences and consequently BC-related mortality, some long-term BC survivors will develop ischemic heart disease (IHD). Since the mid-90s, long-term cardiac morbidities/mortality have been reported after radiotherapy. In 2005, the EBCTCG meta-analysis showed that heart disease significantly increases mortality of patients with BC (hazard ratio: 1.27, p=0.0001) (1). From 2000, the BC radiation oncology community has focused on identifying parameter(s) that predict late cardiac toxicities. It was first reported that the maximal heart distance correlates with the percentage of irradiated heart volume (2). More than a decade later, Darby and colleagues assessed IHD risk in function of the heart exposure and the presence of cardiac risk factors (history of circulatory disease, diabetes, chronic obstructive pulmonary disease, smoking, high body mass index, regular analgesic use) in a population-based case-control study of women with BC who received radiotherapy between 1958 and 2001 and with major coronary events (i.e. myocardial infarction, coronary revascularization or death from IHD) or not (controls) (3). This study relied on real clinical data with estimated radiotherapy plans. From this study, dose-volume histograms, mean doses and equivalent doses delivered in 2Gy fractions (EQD2) were generated for the whole heart and for the left anterior descending (LAD) coronary artery. The mean heart doses (MHD) were 4.9Gy for the whole population and 6.6Gy for patients with left BC, respectively. The mean LAD coronary artery dose and mean EQD2 were 9.9Gy and 4.4Gy for the whole population. The authors concluded that MHD is the most predictive factor of developing a major coronary event, and higher MHD values significantly enhance the risk of major coronary event. Taylor and colleagues assessed MHD predictive value after the introduction of modern radiotherapy techniques (3D conformal and inverse-planned intensity modulated radiation therapy, IMRT) (4). Their treatment plans showed MHD of 9.2Gy for 3D conformal radiotherapy of left-sided BC and internal mammary chain (IMC) and of 8.6Gy for IMRT. These values decreased to 3.4Gy and 5.6Gy with 3D conformal radiotherapy and IMRT, respectively, when the IMC was not included. Furthermore, in patients with unfavorable anatomy (pectus excavatum), MHD was 14.8Gy. The estimated radiation-induced heart disease incidence rates were 1.3% and 2.5% and the cardiac-related mortality rates were 0.6% and 1.2% without and with IMC irradiation, respectively, for 50-year-old patients without any cardiac risk factor, regardless of the radiation technique.

Nevertheless, several studies challenged the use of MHD as an appropriate surrogate parameter. The BACCARAT study recommended assessing the dose distribution of the cardiac substructures, in particular the LAD (5). Recently, Naimi et al. (6) studied the radiation dose distribution to cardiac subvolumes in left breast cancer radiotherapy for 50 patients treated with 3D-conformal hypofractionated radiotherapy. They showed a poor correlation between MHD and dose to cardiac substructures and suggested to define the left ventricle and the LAD as separate organ at risk.

To our knowledge, these observations have not been reported with IMRT techniques. Although the American Society for Radiation Oncology (ASTRO) does not recommend IMRT for the routine delivery of WBI following breast-conserving surgery, some studies showed that IMRT use is increasing worldwide (7–9). Pierce and colleagues recently reported that in the USA, approximately 40% of patients with BC receive IMRT (10). The present study objective was to determine whether MHD is representative of similar heart substructure exposure after 3D or IMRT for BC. To this aim, first we estimated the MHD following breast irradiation by IMRT and using an optimal strategy with a 3D technique. Then, we analyzed the heart and LAD dose distribution with an equivalent MHD (obtained from the radiotherapy plans for the two techniques) and the impact of MHD variations on these structures.

## Materials and methods

After the study approval by the local Ethics Committee, ten patients with left-sided BC and unfavorable cardiac anatomy (i.e. maximum heart depth ≥1.0 cm within the tangent fields) and/or unfavorable anatomy (pectus excavatum) and their relative treatment plans were retrieved from our database (**Figure S1**). These patients were treated between December 2012 and March 2016. All patients had lumpectomy and sentinel node biopsy followed by adjuvant radiotherapy. Due to their unfavorable cardiac anatomy, all patients received IMRT using the RapidArc (RA) technology without breath-hold technique. All patients were on supine position, both arms over the head with personalized foam and underwent non-contrast Computed Tomography (CT)-based simulation (Optima CT580 RT, General Electric Healthcare, Waukesha, WI). CT images were acquired using a 2.5 mm slice thickness from the top of the second cervical vertebral body to the bottom of the first lumbar vertebral body.

### Delineation of target and organs at risk (OAR) volumes

As the BC was removed by surgery, no gross target volume was delineated. The breast Clinical Target Volume (CTV) was defined according to the ESTRO guidelines (11). Briefly, breast CTV encompassed the clinical (delineated by radio-opaque markers) and visible mammary gland. The tumor bed CTV included surgical clips with a 20mm-margin extension (12, 13). Heart and LAD coronary artery (including the LAD coronary artery and interventricular branch) were delineated using the atlas by Feng and colleagues (14). The Planning Target Volume (PTV) was defined as a 3D-expansion of the CTV with a margin of 7mm. All PTV and CTV were limited 5 mm under the skin. The total doses delivered to the breast and tumor bed PTV were 52.2Gy and 63.22Gy in 29 fractions, respectively.

### Treatment plans

Patients were treated according to RA technique owing to their unfavorable cardiac anatomy and/or unfavorable anatomy (pectus excavatum). RA radiotherapy plans were prepared using the mono-isocentric technique with six partial rotation arcs, each with 50° gantry rotations, as described by Tsai et al. (15). Photon Optimizer (PO, v15.5) was used for RA optimization. RA radiotherapy plans were considered as completed when at least 99% of the breast CTV received a total dose of 49.6Gy (i.e. 95% of 52.2Gy) and when at least 95% of the tumor bed PTV received 95% of the total dose of 63.22Gy (SIB technique). OAR dose constraints are summarized in **Table 1**. Dose distributions were calculated with the analytical anisotropic algorithm (v15.5, Varian Medical Systems, Palo Alto, CA, USA) on a TrueBeam linear accelerator equipped with a Varian 120 multileaf collimator.

**Table 1:** Dose constraints for the indicated organs at risk.

OAR	Constraints
Heart	D_1%_< 40 Gy
	D_mean_ < 10 Gy
Left Lung	D_20%_ < 22 Gy
	D_10%_ < 30 Gy
	D_80%_ < 5 Gy
	D_mean_ < 13 Gy
Right Lung	D_1%_ < 10 Gy
	D_mean_ < 5Gy
Right Breast	D_1%_ < 10 Gy
	D_mean_ < 5Gy

For dosimetric comparison, virtual 3D-conformal RT plans were generated using common tangent wedged fields (6MV photon energy; maximum 400MU/min dose rate). A total dose of 52.2Gy in 29 fractions was prescribed to breast PTV following by a boost dose of 11.02Gy in 29 fractions to the tumor bed (equivalent prescription to the RA technique).

*Study A*: All patients had a RA radiotherapy plan to optimize the target volume coverage and to limit OAR exposure, particularly the heart (treatment performed). Then, heart and LAD coronary artery exposure were compared in function of the treatment technique (RA IMRT and virtual 3D conformal radiotherapy). For this, virtual 3D conformal radiotherapy plans were created to ensure that the target volume dose-volume histograms would be the same as those obtained with the RA radiotherapy plans. Heart exposure to high total dose were monitored by calculating the mean dose (Gy) and the volumes at 30, 40 and 45Gy (V30Gy, V40Gy and V45Gy; i.e. the percentage of heart volume in % encompassed by the 15Gy, 30Gy, 40Gy and 45Gy isodose, respectively). Concerning LAD coronary artery exposure, V15Gy, V30Gy, V40Gy and V45Gy were reported.

*Study B*: Based on the individual MHD obtained in the RA radiotherapy plans, virtual 3D conformal radiotherapy plans were generated to obtain similar MHD, regardless of the PTV coverage. Heart and LAD coronary artery exposure were monitored by calculating the V5Gy, V10Gy, V30Gy, V40Gy and V45Gy (percentage of heart volume and LAD coronary artery volume in cc encompassed by the 5Gy, 10Gy, 30Gy, 40Gy and 45Gy isodose, respectively). The aim of this study is to analyze the difference in dose distribution for different dose levels between the two techniques for similar MHD values.

*Study C*: Darby and colleagues reported in their population-based case-control study a MHD of 6.6Gy for left-sided BC (with 3D conformal radiotherapy) and an increase by 7.4% of major coronary events for each 1Gy increment in the MHD. Study C aim was to assess the relationship between the MHD, and the heart volume receiving at least 30Gy, 40Gy and 45Gy in function of each additional 1Gy to the MHD. To this aim, the individual 3D conformal radiotherapy plans from study A were used: MHD was >6.6Gy (from 7.6Gy to 29.7Gy) and <6.6Gy (from 1.4Gy to 4.3Gy) in six and four patients with unfavorable cardiac anatomy, respectively. For each patient, the 3D conformal radiotherapy plan was recalculated to obtain a MHD between 3.5Gy and 7Gy, either by decreasing or increasing the MHD (0.5Gy each time). The percentage of heart volume, and LAD coronary artery volume (in cc) encompassed by the 30Gy, 40Gy and 45Gy isodose were retrieved for each patient. The correlations between MHD and heart volume (percentage) or LAD coronary artery volume (in cc) were analyzed.

### Statistical considerations

Data were described using the mean, minimal and maximal values for continuous parameters and percentages and 95% confidence interval for categorical parameters.

Continuous parameters were compared between categories using the Wilcoxon - Mann Whitney test. Correlation analyses between continuous parameters were performed using the Spearman’s rank correlation coefficient.

## Results

### Patients, OAR volumes

All patients had pT1N0 (n=9) or pT2N0 (n=1) left invasive ductal carcinoma BC. The mean breast CTV was 761.3 cc (min – max, 175 cc – 1642.5 cc), mean heart volume 612cc (min – max, 439 – 749cc) and mean LAD coronary artery volume 2.74 cc (min – max, 1.1 – 5.7 cc).

### Significant decrease of heart and LAD exposure to high dose with RapidArc for the same target coverage (study A)

**Figure 1:**
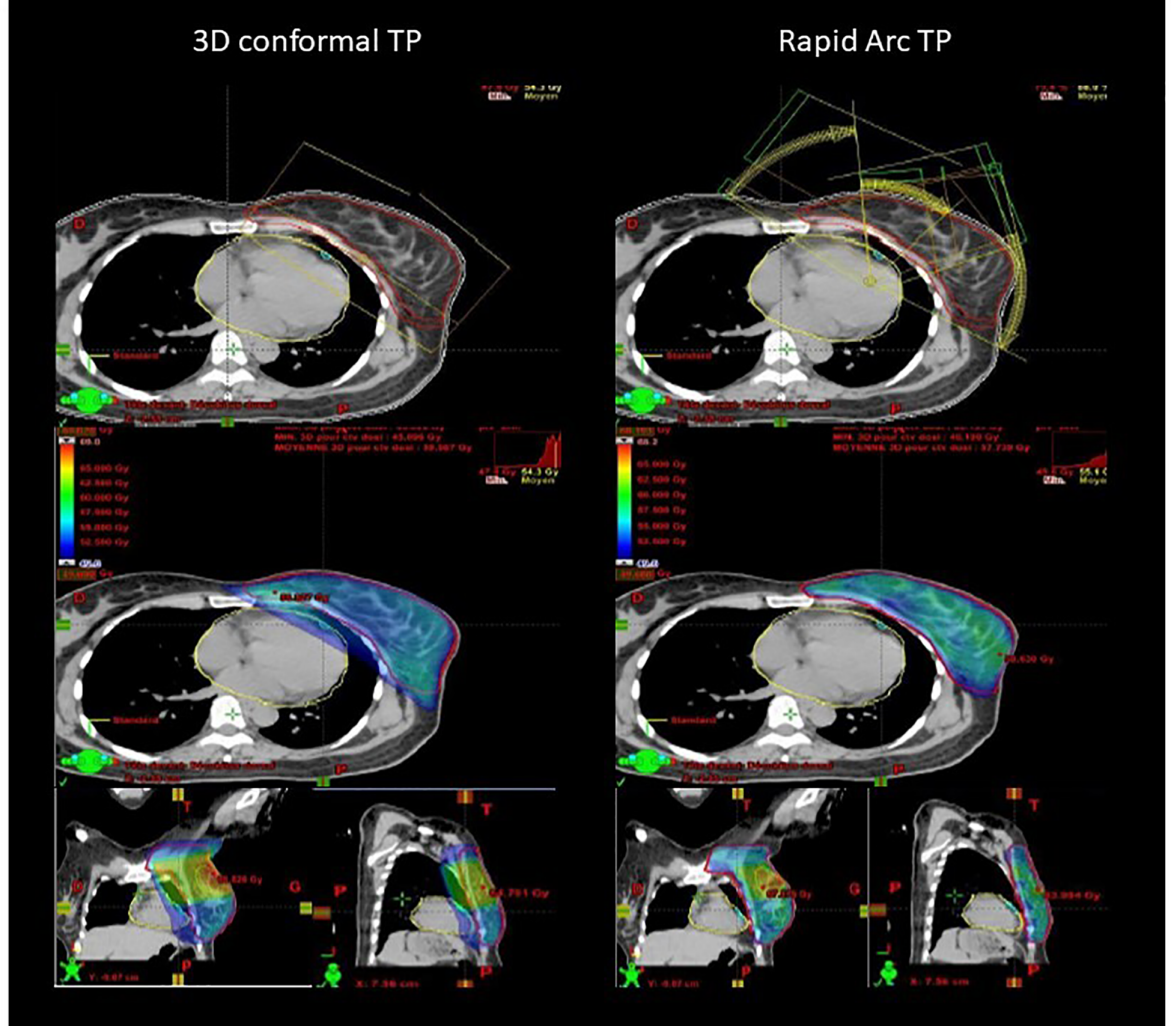
Example of comparison of 3D conformal (virtual) radiotherapy plan with tangent fields and RapidArc-based IMRT (real) plan with six partial rotation arcs to ensure the same target volume coverage (95% isodose displayed).

**Figure 2:**
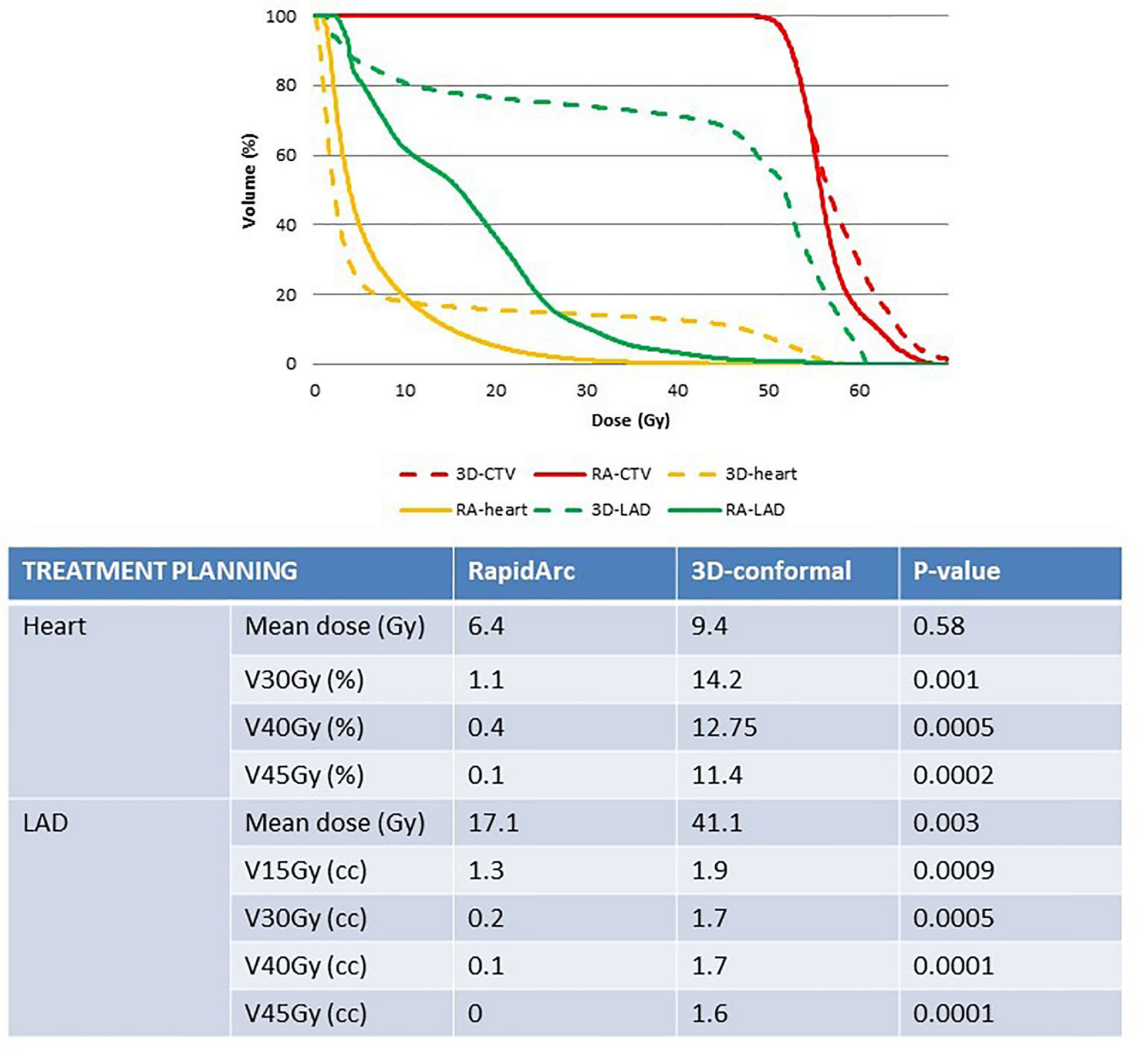
Comparison of heart and LAD exposure in the radiotherapy plans (3D-conformal versus RapidArc-based IMRT) for similar target coverage. 3D, 3D-conformal; RA, RapidArc; CTV, clinical target volume; LAD, left anterior descending coronary artery.

In study A, the real RA radiotherapy plan of each patient was compared to the virtual 3D conformal radiotherapy plan with the same CTV coverage (i.e. at least 99% of breast CTV encompassed by the 95% isodose; **Figure 1**). Concerning heart exposure, MHD was not significantly different in the RA and 3D conformal radiotherapy plans (6.4Gy and 9.4Gy). However, heart exposure to high total doses was significantly different: V45Gy, V40Gy and V30Gy were strongly reduced in the RA radiotherapy plans (V30Gy and V45Gy: 1.1% and 0.1%) (**Figure 2**). The mean LAD coronary artery was significantly lower with RA than with 3D conformal radiotherapy (17.1Gy and 41.1Gy; p-value=0.003) as well as the LAD coronary artery V45Gy, V40Gy,V30Gy and V15Gy (**Figure 2**).

### The mean heart dose is not representative of heart substructure exposure when using RapidArc IMRT (Studies B and C)

**Figure 3:**
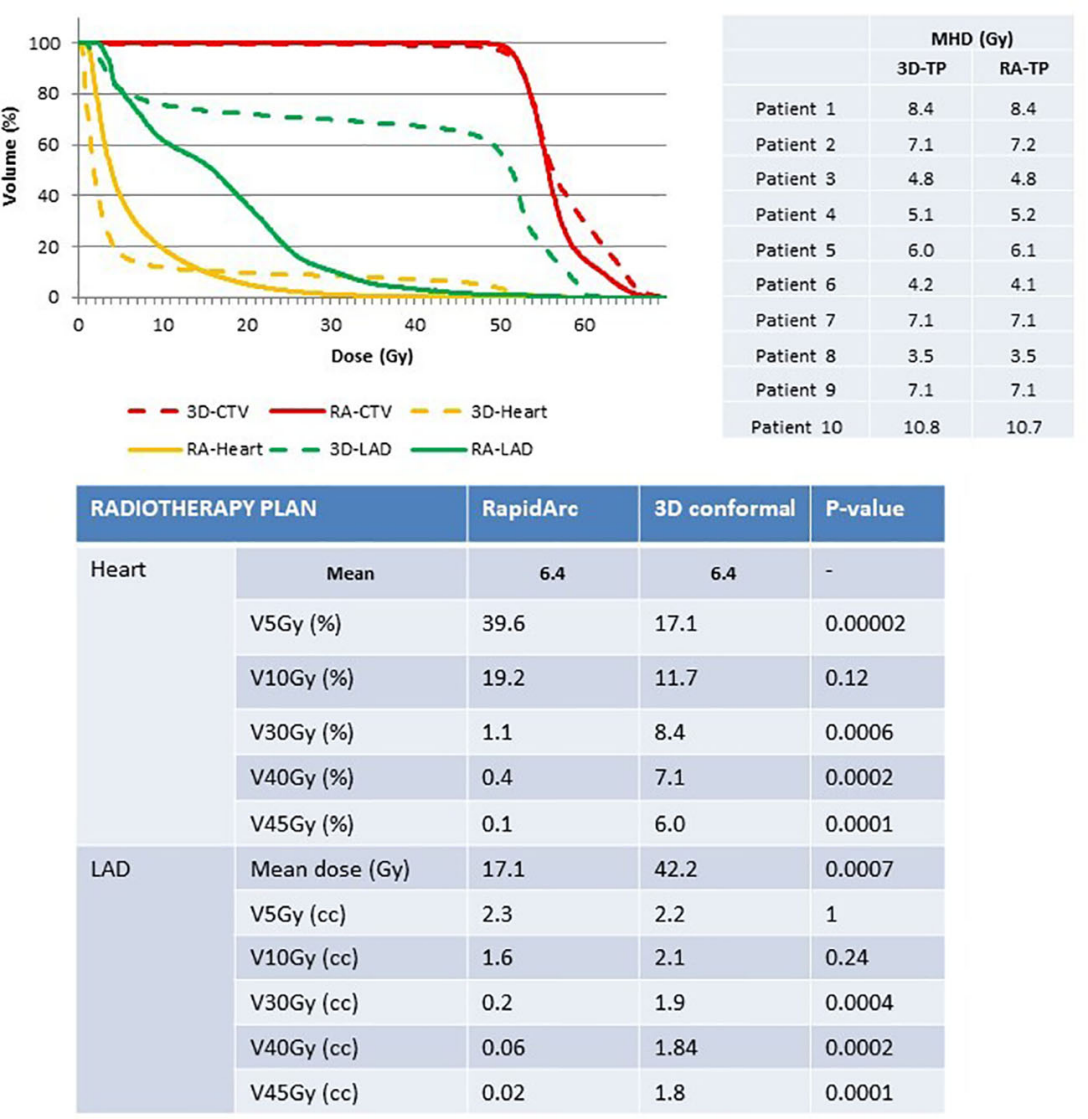
Comparison of heart and LAD exposure in the radiotherapy plans (3D-conformal versus RapidArc-based IMRT) for similar intra-patient MHD. 3D, 3D-conformal; RA, RapidArc; CTV, clinical target volume; LAD, left anterior descending coronary artery; TP, treatment plan.

*Study B.* The intra-patient MHD was similar with both radiotherapy techniques (MHD=6.4Gy), whereas dose distribution was significantly different. A better breast PTV coverage was observed with the RA than with the 3D conformal radiotherapy plans (V49.6Gy=99% *versus* 97.5%). Moreover, heart exposure to high doses was significantly decreased with the RA technique (<1% of heart volume for V30Gy, V40Gy and V45Gy), while it was increased for V5Gy exposure compared with the 3D conformal technique (39.6% versus 17.1%, respectively) (**Figure 3**). The mean LAD coronary artery dose was significantly reduced in the RA compared with the 3D conformal radiotherapy plans (17.1Gy *versus* 42.2Gy). LAD coronary artery exposure to low dose was comparable between techniques, whereas exposure to high doses was significantly reduced in the RA compared with the 3D conformal radiotherapy plans (**Figure 3**).

**Figure 4:**
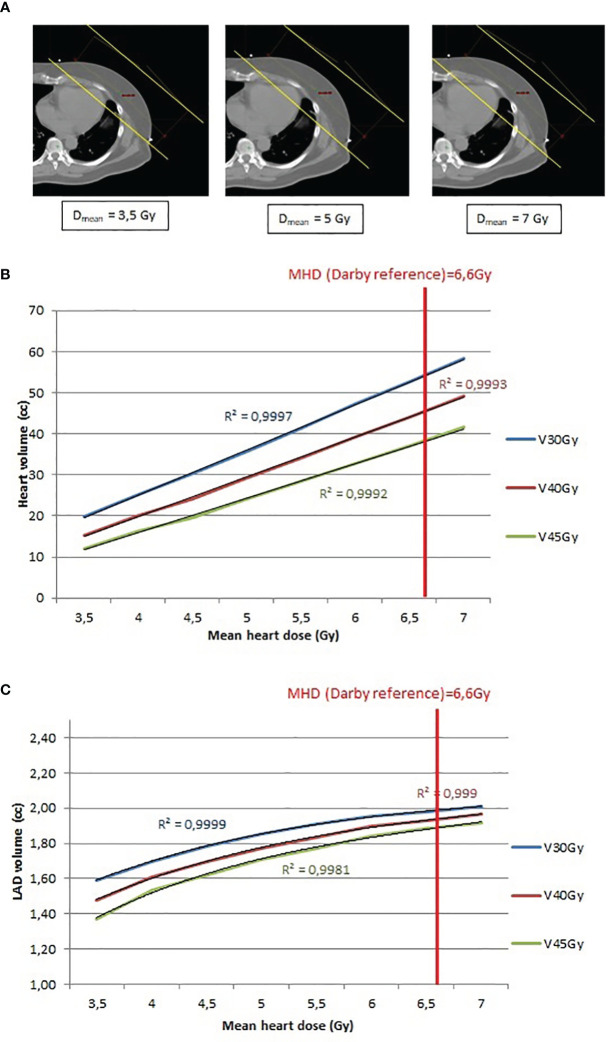
**(A)** Example of 3D tangent fields (yellow lines) plan recalculation to obtain a MHD between 3.5Gy and 7Gy, by step of 0.5Gy. **(B)** Relationship between heart volume (cc) and mean heart dose (Gy) and determination of the R^2^ value. **(C)** Relationship between LAD coronary artery volume (cc) and mean heart dose (Gy) and determination of the R^2^ value.

Study C. The relationship between the MHD, and the heart volume exposure was evaluated in function of each additional 0.5Gy to the MHD (**Figure 4A**). Study C. MHD and heart exposure (V30Gy, V40Gy and V45Gy) showed a strong linear correlation (R^2^ closed to 1) (**Figure 4B**) whereas LAD coronary artery exposure (V30Gy, V40Gy and V45Gy) displayed a polynomial correlation with MHD (**Figure 4C**). When these correlations were assessed for a MHD of 6.6Gy (the reference from the article by Darby et al), the 3D conformal radiotherapy plans achieved heart V30Gy, V40Gy and V45Gy of 8.17%, 6.86% and 5.72%, respectively, whereas the RA radiotherapy plans significantly decreased the heart V30Gy, V40Gy and V45Gy (1.11%; 0.36% and 0.09%, respectively). Similar results were obtained for the LAD coronary artery V30Gy, V40Gy and V45Gy (1.98cc; 1.91cc and 1.87cc, respectively, in the 3D conformal radiotherapy plans, and 0.2cc; 0.06cc and 0.02cc, respectively, in the RA radiotherapy plans).

## Discussion

The present study showed that the use of RA allow a significant better PTV coverage, a significant lesser LAD exposure than 3D-conformal technique in patients with left-sided BC and unfavorable cardiac anatomy and/or unfavorable anatomy. Regarding MHD after RA planning, our results are consistent with those reported by Taylor and colleagues (4) (MHD pectus excavatum=14.8 Gy; MHD unfavorable anatomy=7.1Gy).

The effect on the heart of dose sparing strategies, particularly the role of the deep inspiration breath hold technique, has been extensively studied, by assuming that the MHD is the appropriate dosimetric parameter to evaluate the risk of late cardiac toxicity occurrence.

The reference study by Darby and colleagues showed that the MHD in patients with left-sided BC treated by 2D or 3D conformal radiotherapy should be lower than 6.6Gy (3). The methodology used in this study is one of its limitations: MHD was estimated to be 4.9Gy for a woman with typical anatomy because no individual data was available. In a more recent study based on individual data, the MHD was 4.4Gy when only the left-sided BC was irradiated (without any node field) (16). This study on 910 patients with BC showed that the relationship between acute coronary events and the left ventricle volume receiving 5Gy was more important than MHD. Furthermore, more and more radiation oncologists use IMRT for BC treatment, and the systematic review (studies from 2003 to 2013) by Taylor et al. found that the recommended value from the study by Darby et al. could not be respected when the IMC was included in the radiotherapy prescription (MHD=9.2Gy), and when using IMRT (MHD=8.6Gy) (4).

Our study found that the MHD is not the most appropriate dosimetric parameter for IMRT if we follow clinical rule defined for 3D CRT treatment: heart and LAD coronary artery dose distribution are significantly different in function of the radiotherapy technique (IMRT and 3D conformal radiotherapy), despite comparable MHD. In 3D technique, the MHD value is the result of a small volume receiving a high dose (upper 40Gy) combined with a very large volume of heart not irradiated. It is clear that there is a relationship between the portion of heart irradiated and the toxicity, but these last come from high dose region and the representation of a mean value is not the better way to represent it. Looking the volume of heart receiving dose upper 40 Gy is more representative to the real dose distribution and despite a generally higher D_mean_ with IMRT in clinical routine, the volume irradiated at high dose is significantly lower than in 3D CRT. This is clearly show in the study B for an equivalent MHD for the two techniques. Looking to 3D treatment in our study we try to convert a MHD value leading to clinical effects (as in Darby paper) into a portion of heart irradiated to high volume to define some limits for our optimization in inverse planning techniques (**Figure 3**).

Similarly results were reported by the BACCARAT study (ClinicalTrials.gov: NCT02605512) the aim of which was to determine predictive factors (circulating biomarkers and heart dosimetric parameters) of early radiation-induced subclinical cardiac dysfunction in patients with BC treated by 3D conformal radiotherapy (5). In this study, the MHD and mean LAD coronary artery dose were 2.9Gy and 15.7Gy, respectively. The authors observed that in patients with left-sided BC, the correlation between MHD and left ventricle exposure was stronger (R^2 =^ 0.78) than between MHD and LAD coronary artery dose (R^2 =^ 0.67). Unlike the BACCARAT study, we found a strong polynomial (and not linear) correlation between MHD and LAD coronary artery exposure when using 3D conformal radiotherapy: with increasing doses to the heart, LAD coronary artery exposure progressively increased until it reached a threshold corresponding to the proportion of LAD within the tangent fields. To date, all studies on dosimetric parameters as predictive factors of radiation-induced cardiotoxicity used 3D conformal radiotherapy plan data. Due to the lack of long-term data on IMRT use in patients with BC and the risk of IHD occurrence in such patients, 3D-conformal radiotherapy planning constraints are routinely applied to IMRT treatment planning. The recent study by Loap and colleagues suggested similar observations (17): among the many cardiotoxicity predictive factors found in the literature (mean dose, maximum dose, V3Gy, V5Gy, V10Gy, V15Gy, D90% and D95%), IMRT MHD is not representative of the cardiac substructure exposure in the same way as 3D CRT.

Darby et al. report an increase by 7.4% of major coronary events for each 1Gy increment in the MHD, but we show in our study C that a small increase in the MHD value for 3D CRT technique is generated by a large increase of the heart volume receiving high dose leading to the correlation between the volume of heart irradiated to *dose upper* 40 Gy with toxicity. Evolution of the mean dose in IMRT/VMAT treatment is not lead by the same dose distribution but more with low dose area.

Similar findings were recently reported for lung cancer. In a large retrospective cohort, LAD coronary artery dose exposure was related to severe adverse cardiac events, especially in patients without any history of coronary heart disease. IHD risk (HR=24.8) was significantly higher for patients with LAD coronary artery V15Gy ≥10% without but not with coronary heart disease history (18, 19). Moreover, a mean total coronary artery dose ≥7Gy increased the absolute risk of IHD in patients without coronary heart disease history by 5% in 1 year.

Here, we showed that MHD is not representative of the cardiac exposure: heart and LAD coronary artery dose distribution were significantly different in function of the radiotherapy technique, although MHD was the same, and IMRT significantly decreased heart and LAD coronary artery exposure to high dose. This indicates that using 3D conformal dosimetric constraints for IMRT is not the most appropriate strategy. Future studies are needed to correlate heart and its substructures exposure with prospective clinical data in order to generate appropriate surrogate in cardio-oncology field.

Last, a Sweden study using a national cardiac register assessed the long-term risk of IHD (defined as angina pectoris, acute myocardial infarction, complications due to myocardial infarction, and chronic IHD) after adjuvant radiotherapy, and observed that node irradiation (HR=1.46), post-mastectomy radiotherapy (HR=1.25), and the combination of endocrine therapies and chemotherapy (HR=1.35) increased the IHD risk in patients with left-sided BC (20). The authors reported high LAD coronary artery exposure (mean dose to the distal LAD coronary artery of 26.7Gy) and recommended to include LAD coronary artery radiation dose in radiotherapy plans, with the lowest possible dose. Minimizing the dose to the LAD coronary artery should decrease the risk of later radiation-induced stenosis.

## Conclusion

IMRT techniques significantly reduce high dose exposure to OAR, while, in the meantime, spreading low dose to OAR compared to 3D techniques (where dose distribution is restricted within treatment fields). Even though clinical heart event after BC is clearly correlated with the irradiation of the heart, we show that MHD issue from the cohort of patients irradiated with 3D technique is not directly transposable to modern radiotherapy delivery like IMRT or VMAT. Volume of heart (or substructures) receiving high dose region is certainly a better surrogate.

## Data availability statement

The original contributions presented in the study are included in the article/**Supplementary Material**. Further inquiries can be directed to the corresponding authors.

## Author contributions

CB, JP, and PF contribute to the conception, the design of the work; JP, PF, and SG to the acquisition, and analysis; CB, JP, and PF to the interpretation of data; all authors have drafted the work or substantively revised it. Each author has approved the submitted version and agreed both to be personally accountable for the author’s own contributions.

## Supplementary material

Supplementary Figure 1 CT images showing the anatomy of the ten patients with left breast cancer included in this study. Patients had unfavorable cardiac anatomy (i.e. maximum heart depth ≥1.0 cm within the tangent fields; patients n=9) and/or unfavorable anatomy (pectus excavatum; patients n=1).
