# Supplementary figures and images for: Different meaning of the mean heart dose between 3D-CRT and IMRT for breast cancer radiotherapy
Source: Front Oncol. 2023 Jan 16;12:1066915. doi: 10.3389/fonc.2022.1066915 (PMC9886087; doi:10.3389/fonc.2022.1066915)

Patient 1

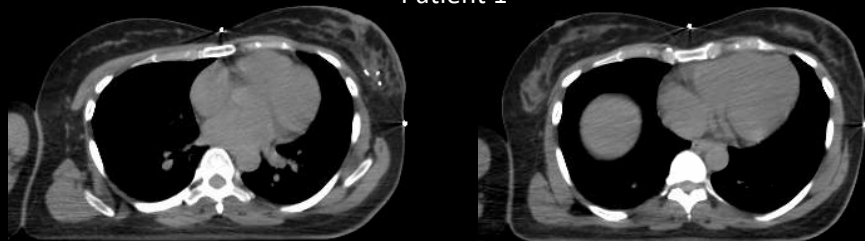

Patient 2

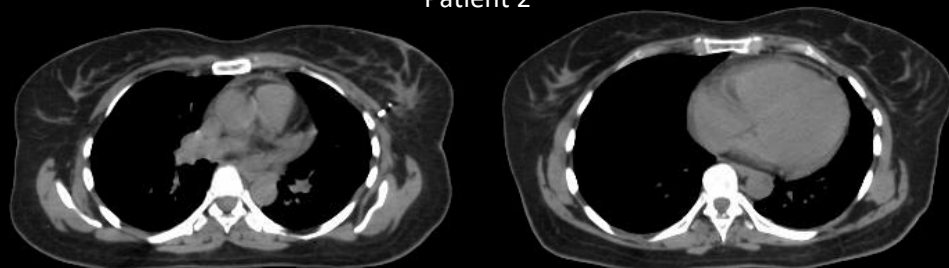

Patient 3

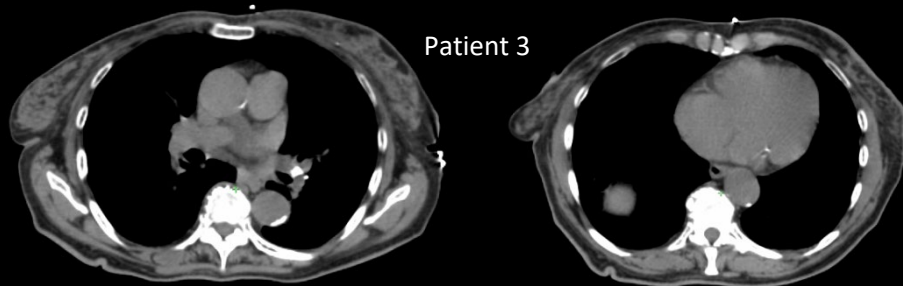

Patient 4

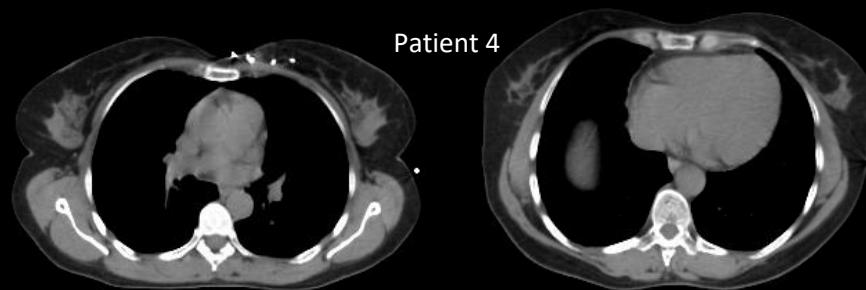

Patient 5

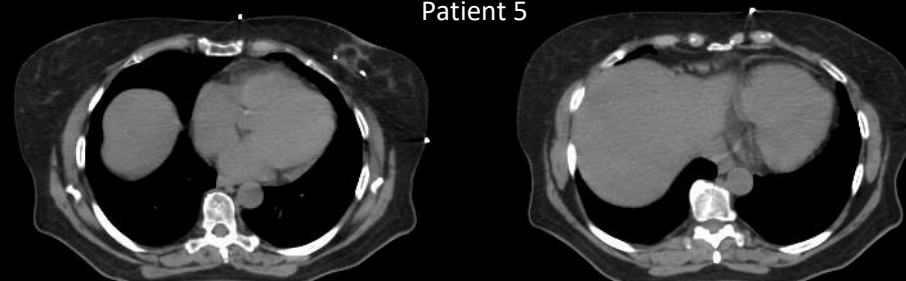

Patient 6

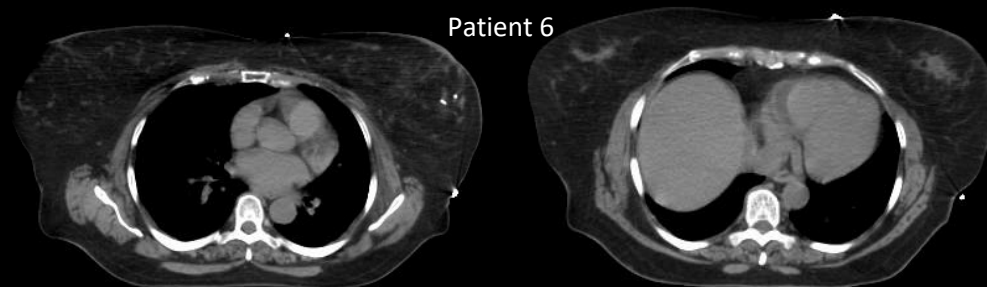

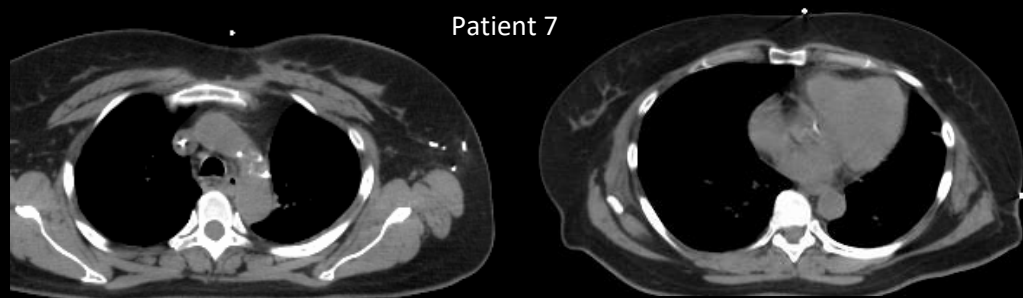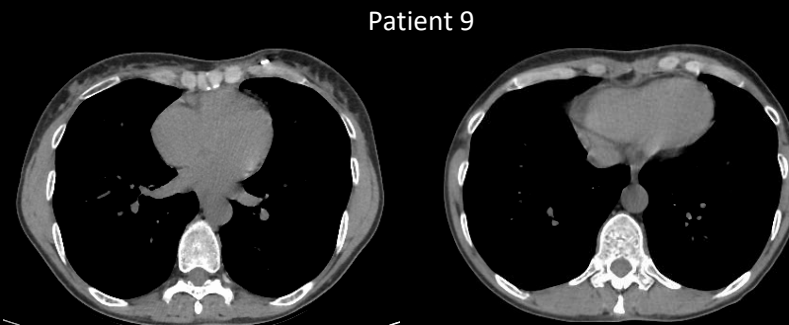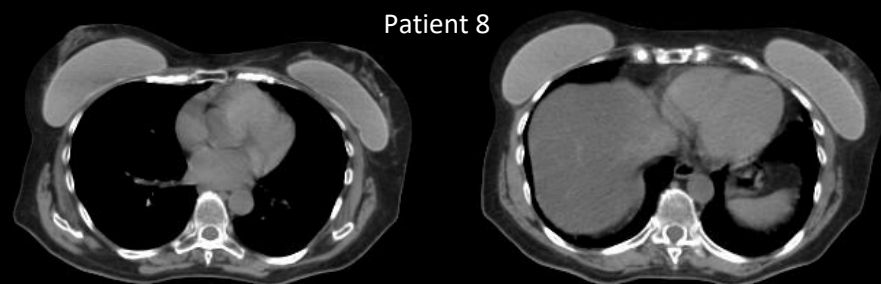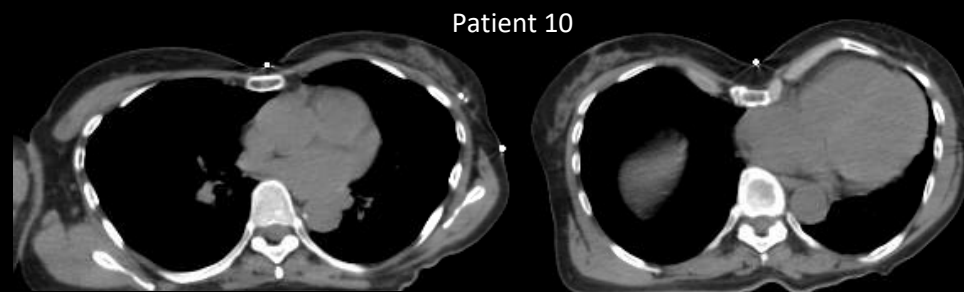

Supplement: Supplementary Figure 1 — CT images showing the anatomy of the ten patients with left breast cancer included in this study. Patients had unfavorable cardiac anatomy (i.e. maximum heart depth ≥1.0 cm within the tangent fields; patients n=9) and/or unfavorable anatomy (pectus excavatum; patients n=1). [file Image_1.pdf]
